# Assessment of Retained Austenite in Fine Grained Inductive Heat Treated Spring Steel

**DOI:** 10.3390/ma12244063

**Published:** 2019-12-05

**Authors:** Anna Olina, Miroslav Píška, Martin Petrenec, Charles Hervoches, Přemysl Beran, Jiří Pechoušek, Petr Král

**Affiliations:** 1 Department of Manufacturing Technology, Faculty of Mechanical Engineering, Brno University of Technology, Technicka 2, 61669 Brno, Czech Republic; piska@fme.vutbr.cz (M.P.); mpetrenec@gmail.com (M.P.); 2 Nuclear Physics Institute of the CAS, Řež 130, 25068 Řež, Czech Republic; hervoches@ujf.cas.cz (C.H.); premysl.beran@esss.se (P.B.); 3 European Spallation Source ERIC, Box 176, 22100 Lund, Sweden; 4 Department of Experimental Physics, Faculty of Science, Palacky University in Olomouc, 17. Listopadu 12, 77900 Olomouc, Czech Republic; jiri.pechousek@upol.cz; 5 Institute of Physics of Materials, Czech Academy of Sciences, Zizkova 22, 61662 Brno, Czech Republic; pkral@ipm.cz

**Keywords:** spring steel, heat treatment, retained austenite, Mössbauer spectroscopy, neutron diffraction

## Abstract

Advanced thermomechanical hot rolling is becoming a widely used technology for the production of fine-grained spring steel. Different rapid phase transformations during the inductive heat treatment of such steel causes the inhomogeneous mixture of martensitic, bainitic, and austenitic phases that affects the service properties of the steel. An important task is to assess the amount of retained austenite and its distribution over the cross-section of the inductive quenched and tempered wire in order to evaluate the mechanical properties of the material. Three different analytical methods were used for the comparative quantitative assessment of the amount of retained austenite in both the core and rim areas of the sample cross-section: neutron diffraction—for the bulk of the material, Mössbauer spectroscopy—for measurement in a surface layer, and the metallographic investigations carried by the EBSD. The methods confirmed the excessive amount of retained austenite in the core area that could negatively affect the plasticity of the material.

## 1. Introduction

The modern production of environmentally friendly vehicles has imparted progress in the automotive industry, leading to the development of many innovative features. The main impacts are fuel savings, which can be provided by reducing the weight of vehicles, for example, by replacing the contemporary wiring with innovative copper-aluminum clad composite wires [1,2], by using modern lightweight construction materials [3], or by introducing electric automobiles [4].

Due to the electric car strategy, new requirements are also given to the chassis components in terms of higher strength (above 2100 N/mm^2^) and sufficient toughness. Several approaches could be taken in order to obtain the required results. For example, an application of the optimized treatment with the implementation of a new processing technology, which can preferably be done via the methods for applying severe plastic deformation (SPD), such as equal channel angular pressing (ECAP) [5,6], accumulative roll bonding (ARB) [7], or rotary swaging [8,9]. The advanced treatments contribute to the optimization of the final mechanical properties via mechanical mixing and substantial grain refinement [10].

Another approach is to develop thermo-mechanically rolled fine-grained steel with a mixed microstructure consisting of martensite and/or bainite and a considerable amount of stabilized retained austenite (RA) [11,12]. In the last few years, many reports about the development of quenching and partitioning (Q&P) processes for the production of heat-treated steel with the optimal combination of high strength and ductility [13,14,15,16,17,18,19] can be found. All studies emphasize the key role of RA and its morphology, size, and distribution. The stability of RA also plays a key role in the prevention of tempered martensite embrittlement caused by the decomposition of RA to cementite and ferrite [20].

Grain refinement of steel microstructures was also reported to improve the stability of retained austenite by decreasing the size of blocks of RA [21]. Thermo-mechanical rolling is used to obtain the fine grain structure of the hot rolled wire products [22], along with the vanadium and/or niobium micro alloying of steel [23,24]. Vanadium micro-alloying also suppresses the growth of austenite grains under higher temperatures up to 1000 °C during austenitization in comparison with V-free spring steel [25,26,27], which contributes to higher strength of the steel after heat treatment.

Based on the above mentioned, the vanadium micro-alloying spring steel is supposed to provide an excellent combination of strength and ductility under optimal parameters of heat treatment. At the same time, the amount of RA and its distribution in the structure should be carefully studied because RA could be a key factor in the production of the heat-treated steel with advanced properties. Hence, the precise and reliable methods of RA assessment should be implemented.

## 2. Experimental Material and Methods

### 2.1. Material

**Figure 1:**
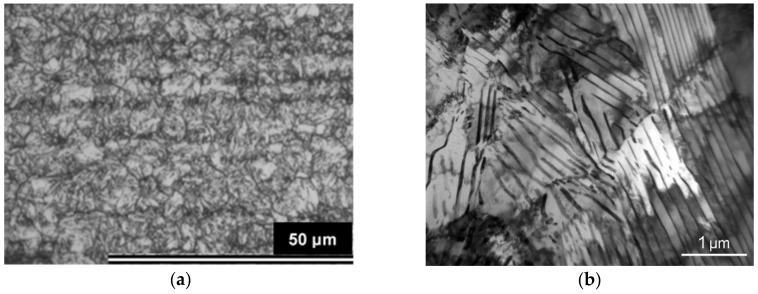
Austenite grain size and microstructure of hot rolled wire: (**a**) austenite grain size G13.0 according to ASTM E112; (**b**) cementite lamellae (transmission electron microscopy).

A wire rod of vanadium micro-alloyed 0.6C-0.6Mn-1.4Si-0.6Cr (wt.%) spring steel was investigated in this study. The chemical composition of the steel is presented in Table 1. A wire rod with a diameter of 17.00 mm was produced by thermo-mechanical hot rolling and was cold drawn to a diameter of 15.50 mm preceding inductive heat treatment. The microstructure of hot rolled wire rod consisted of a fine grain perlite-ferrite structure with a cementite lamellae of 17 nm and former austenite grain size of G13.0 (an average grain diameter of 3.3 µm) according to ASTM E112 (Figure 1). The tensile mechanical properties of hot rolled wire were: ultimate tensile strength (UTS) 1350 N/mm^2^ with a reduction of area value of (Z) 46%.

Parameters of the induction heat treatment are presented in Table 2. All specimens were inductively heated up to an austenitization temperature 850 °C. Then specimens were quenched by water spraying (Q1–Q4 specimens) to the specified temperatures. Specimens Q1 and Q2 were quenched to 40 °C and specimens Q3 and Q4 were quenched 100 °C below calculated martensite start temperature $M_{S}={280\;}^{{}^{\circ}}C$ (Equation (1)) [28]. Temperature *M_f_* was estimated based on [29] as −50 °C with respect to the carbon content in the steel.

Finally, specimens were inductively tempered at three different temperatures (Table 2) and cooled by water spraying until the ambient temperature (QT1–QT4 specimens). Tempering temperatures 460 °C and 300 °C were chosen as the limit values for the heat treatment of silicon alloying steel grades [20], and tempering temperature 420 °C was set in order to improve the strength characteristics of the heat-treated spring steel.

It should be additionally mentioned that measurements of quenched specimens Q3 and Q4 were considered as informative ones. Structural characteristics of these samples were affected by the slow air cooling until ambient temperature after the interrupted quenching by water spraying to 180 °C before the measurements, as the direct investigation under 180 °C required comprehensive in situ study. At the same time, measurements of QT3 and QT4 specimens fully corresponded to the investigated heat treatment parameters as they were tempered immediately after reducing to 180 °C.

(1) $$M_{S}({}^{\circ}C)=521-353C-225Si-24.3Mn-27.4Ni-17.7Cr-25.8Mo$$

Mechanical properties of the specimens were measured by means of the tensile test machine WPM UPC 1200 for quenched and tempered conditions. Hardness maps for all specimens were obtained by means of semi-automatic micro hardness equipment Durascan (Struers, Ballerup, Denmark), method HV1.

Then, three different analytical methods were used in order to characterize the obtained microstructures of specimens after heat treatment. The main focus was given to the amount of RA, its distribution, and its effect on the mechanical properties of steel.

### 2.2. Neutron Diffraction

The first method was the neutron powder diffraction (ND), which provided the average information from the whole bulk of the material. The room temperature diffraction patterns were collected on the MEREDIT instrument (Nuclear Physics Institute, Řež, Czech Republic) [30] at the Nuclear Physics Institute in Rez near Prague [31]. The mosaic copper monochromator (planes (220)) (Nuclear Physics Institute, Řež, Czech Republic) was used to provide neutrons with a wavelength of 1.46 Å. Data were collected between 4° and 144° of 2θ with a step of 0.08° 2θ. The samples were rotated along the vertical axis during measurement to minimize the influence of the texture and the preferred orientation on the phase fraction analysis. The full pattern structural refinements were performed using the program FullProf (Version 6.30 - Sep2018, The FullProf Suite, France) [32]. For the microstructural analysis, the instrument profile was obtained by fitting the diffraction pattern of the standard SiO_2_ powder sample collected at the same conditions. Two sets of samples were investigated. The first set contained the initial cylinders of as-prepared heat-treated specimens with a diameter of 15.5 mm. The second set was manufactured from the first one after the ND measurements by removing the rim part. Only the core of the original cylindrical specimens with a square profile and dimensions of 7.5 × 7.5 mm^2^ was used. The 15 mm length of the specimens was kept constant for all the measurements.

### 2.3. Mössbauer Spectroscopy

Mössbauer spectroscopy is a nuclear resonance spectroscopic technique based on the physical phenomenon of recoilless nuclear emission and resonant absorption of gamma rays [33]. This experimental technique provides qualitative and quantitative analysis of materials (e.g., structural, phase, and magnetic information) containing specific elements. The ^57^Fe isotope shows the most favorable parameters for Mössbauer spectroscopy. The backscattering geometry allows us to analyze surfaces of bulk materials. Hence, ^57^Fe Mössbauer spectroscopy has become a very important experimental method in steel characterization. In this study, scattering method utilized the conversion X-rays registration (the conversion X-rays Mössbauer spectroscopy—CXMS), which analyzes material surface up to depths of 1–20 µm. 

Nuclear hyperfine interactions can be analyzed by means of Mössbauer spectroscopy [34]. The hyperfine parameters are isomer shift, quadrupole splitting, and hyperfine magnetic splitting. The isomer shift is a result of the Coulomb interaction between the nuclear/nuclei charge and the electron charge [35]. The charges distributed asymmetrically around the atomic nucleus (electrons, ions, and dipoles) increase the electric field gradient, which differs from zero on the site of nucleus. These electric quadrupole interactions cause a splitting of the excited nuclear level [35] and provide the information about bond properties and the local symmetry of iron site. [33,34,36]. The third hyperfine parameter is magnetic splitting. This magnetic field can originate within the atom itself, within crystals via exchange interactions, or it can be external one. The magnetic field (nuclear Zeeman effect) splits the nuclear states [35].

The iron-phases analysis of the specimens was established by means of Mössbauer Spectroscopy [37]. Polished non-etched metallographic cross-sections were prepared for all specimens. The measurements were performed in a backscattering geometry at room temperature with a ^57^Co(Rh) source, and spectra were recorded up to 512 channels. The isomer shift referred to the calibration of the alpha-iron sample at room temperature. The Mössbauer spectra fitting procedure was carried out using MossWinn (version 4.0, Author Dr. Zoltán Klencsár, Budapest, Hungary) [38] software. Paramagnetic, γ-Fe, and RA phases were fitted by one singlet. Magnetically ordered phases (ferrites, α-Fe, and those in the martensitic structure) represented by sextet components were fitted with a magnetic hyperfine field distribution [37]. The fitting process was set as free for all parameters of the Mössbauer spectra.

### 2.4. Electron Backscatter Diffraction (EBSD) Analysis

The same cross-sections then were additionally used for EBSD analysis of the specimens Q1–Q4 in order to obtain the information about phase distribution within the rim and core areas of the specimens. Samples for EBSD were mechanically ground using SiC papers up to 4000 grit, and were polished using colloidal silica with a 0.06 μm particle size. Finally, the specimens were electropolished using 900 mL acetic acid and 100 mL perchloric acid at 20 °C for 10 s in order to remove the strains induced by mechanical preparation. The microstructures were investigated by scanning electron microscope Tescan Lyra 3 equipped with a NordlysNano detector operating at an accelerating voltage of 20 kV with the specimen tilted at 70°. The EBSD data were analyzed using HKL Channel 5 (version 5.11.10405.0, Oxford Instruments plc, Abingdon, United Kingdom) software developed by Oxford Instruments.

## 3. Results and Discussion

### 3.1. Mechanical Properties

**Figure 2:**
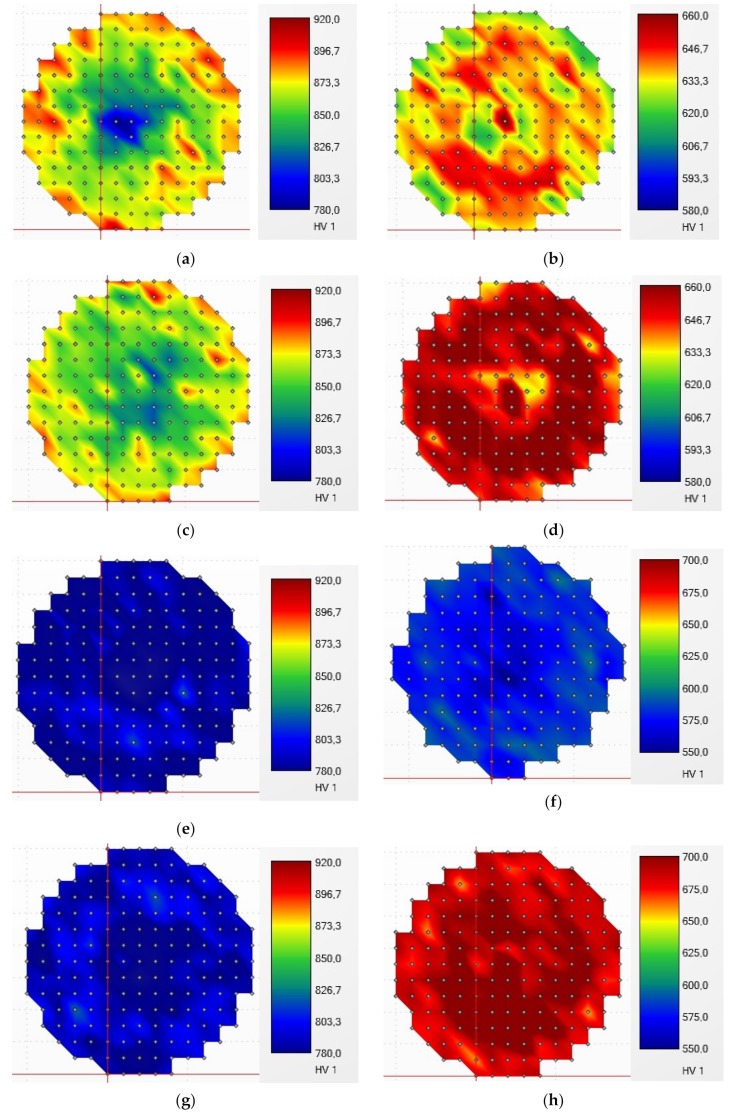
Hardness mapping of the specimens, method HV1: (**a**) Q1; (**b**) QT1; (**c**) Q2; (**d**) QT2; (**e**) Q3; (**f**) QT3; (**g**) Q4; (**h**) QT4.

In order to visualize the inhomogeneity of the microstructure after fast induction heating, the hardness maps were measured for all specimens (Figure 2). Average hardness values for all specimens Q1–Q4 and QT1–QT4, along with the results of the tensile test, are shown in Table 3.

**Figure 3:**
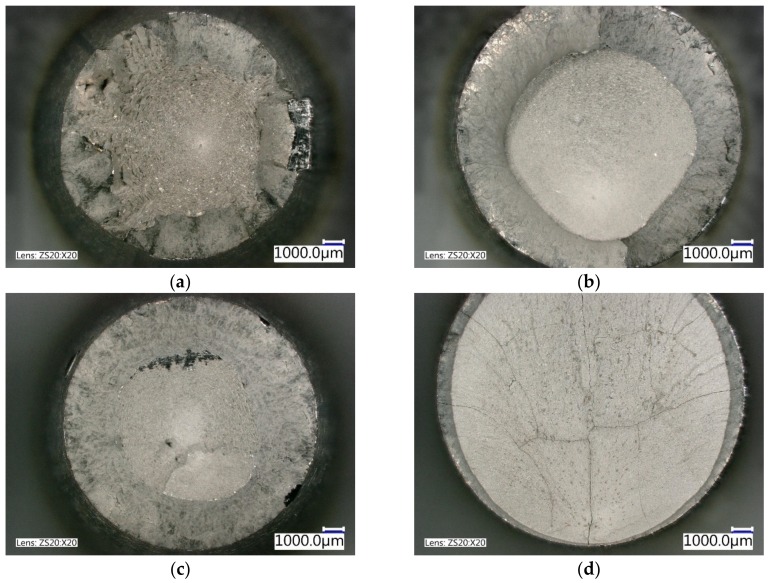
Fracture surface of the QT specimens: (**a**) QT1—cup and cone pattern; (**b**) QT2—non-round cup and cone pattern; (**c**) QT3—cup and cone pattern; (**d**) QT4—brittle fracture.

Based on the results of the tensile test, only samples QT1 and QT3 had the cup and cone fracture pattern after the tensile test (Figure 3a,c) and could be used for the production of chassis components. For specimen QT2 (Figure 3b), it had a non-round cup and a cone pattern, and specimen QT4 had a brittle fracture pattern (Figure 3d), which indicates the low plasticity of these specimens; the suggested schemes of the heat treatment (QT2 and QT4) with lower tempering temperatures are not appropriate for the production of the chassis components with advanced properties.

### 3.2. Neutron Diffraction

**Figure 4:**
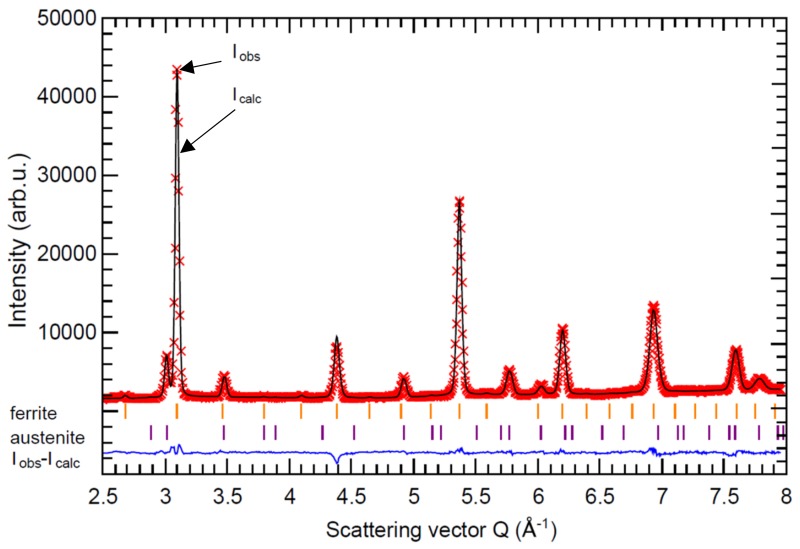
Measured (red crosses) and calculated (black line) neutron diffraction pattern of the specimen QT3 together with their difference (blue bottom line). Small vertical bars represent the Bragg positions for ferrite and austenite, respectively.

The neutron powder diffraction technique has an advantage for when the necessity of the average structural and microstructural information from a large volume is needed. All collected neutron diffraction patterns showed the presence of two crystallographic phases. An example of a measured and calculated neutron diffraction pattern of the specimen QT3 is presented in Figure 4. The minor phase reflections can be indexed as a face-centered cubic (FCC) lattice with a cell parameter of about 3.58 Å. This phase was recognized as RA. The major phase reflections could be within the first approximation indexed as a body-centered cubic (BCC) lattice with a cell parameter of about 2.87 Å, which looked like a fingerprint of the ferrite phase. From the crystallographic point of view, there was no possibility to distinguish a difference between ferrite and low-carbon α-martensite phase. α-martensite had the same lattice as ferrite, but due to the high dislocation and defect density, the strong microstructural reflection broadening or asymmetry could be observed, but the quantitative evaluation of the phase ration was not possible.

The detailed analysis of the neutron diffraction profile showed the significant reflection broadening of both phases surpassing the instrument broadening (significant specimen contribution). It indicates the presence of an increased microstrain in the specimens. In addition, in the case of the specimens Q1–Q4, the shape of the reflections of the ferrite phase was found to be strongly asymmetric, indicating the split of the cell parameters and the decrease of the symmetry from cubic to tetragonal. The appearance of the tetragonality indicates the presence of the martensitic phase. The results of the structural and microstructural analysis are depicted below.

**Figure 5:**
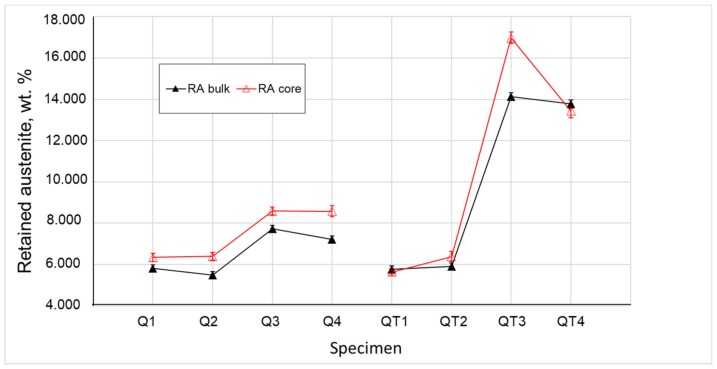
Weight fraction of retained austenite in (%).

The amount of RA calculated from the full pattern fitting is presented in Figure 5 as a function of quenching and tempering temperatures, i.e., parameters of the heat treatment. The comparison of the measurements of the whole specimen and the core area confirmed that the excessive amount of RA was located at the specimen center. The reason for the higher amount of RA in the core area was possibly the relatively slow heat transfer from the surface to the core during the quenching process, which leads to the significantly slower and delayed transformation within the specimen core.

In order to evaluate the influence of RA on the mechanical properties of the material, specimens QT1 and QT3 should be compared. The amount of RA in the bulk of the specimen QT1 is 2.4 times lower than in QT3, but both samples showed the reduction of area ≥35%, which is acceptable for the production of chassis components. It indicates that ductility is not affected by the higher amount of stabilized RA in the QT3 specimen, and it reaches similar values for both QT1 and QT3 specimens. Contrarily, UTS and hardness show a significant decrease for the QT3 specimen with the higher amount of the soft FCC (RA) phase.

**Figure 6:**
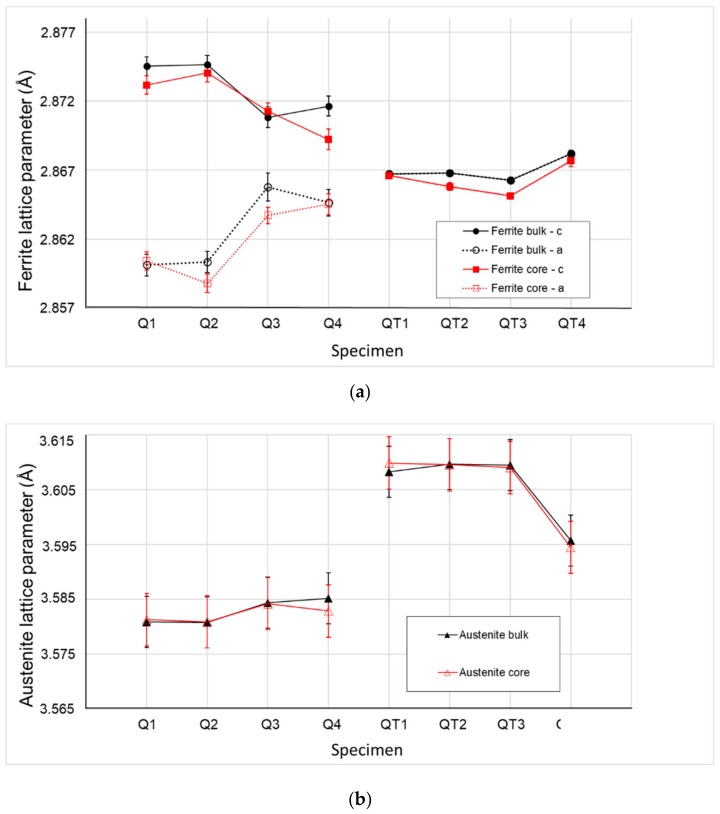
Lattice parameters: (**a**) body-centered cubic – ferrite; (**b**) face-centered cubic – austenite.

The change in lattice parameter for ferrite/α-martensite and austenite, which perfectly describes its evolution with respect to the different parameters of heat treatment, can be seen in Figure 6. As the speed of induction heating was considered to be constant for all samples, it can be concluded that the evolution of the lattice parameter was attributed to the different temperatures and, therefore, to the carbon activity and its diffusion under such temperatures. The highest value of tetragonality, i.e., the c/a parameter, was observed for quenched specimens Q1 and Q2 (1.008 in the bulk), which confirmed the high amount of martensitic phase in the structure (Figure 6) [39]. On the contrary, the tetragonality of all QT specimens was zero, which suggests that the tempering temperature was sufficient for carbon diffusion from BCC lattice.

The lattice parameter for the FCC phase was the opposite of the BCC evolution. Obtained results also correspond with the description of phase transformation in the TRIP C-Mn-Si sheet steel studied by Yu et al. [40] by means of in situ neutron diffraction. Yu et al. attributed the expansion of the lattice parameter of FCC to the carbon enrichment of RA, and expected the shrinkage of FCC lattice after further tempering above 470 °C due to diffusion of the carbon from RA to ferrite.

**Figure 7:**
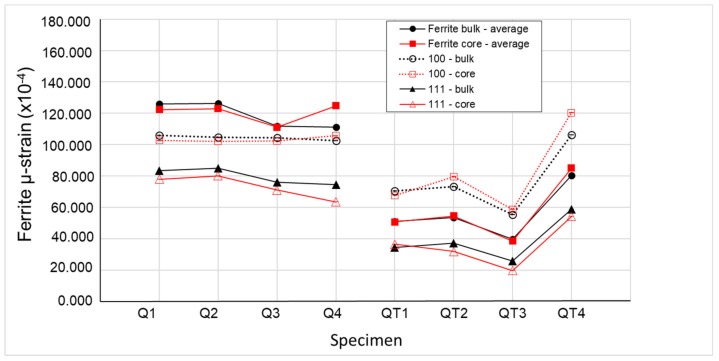
Distribution of µ-strain in BCC.

**Figure 8:**
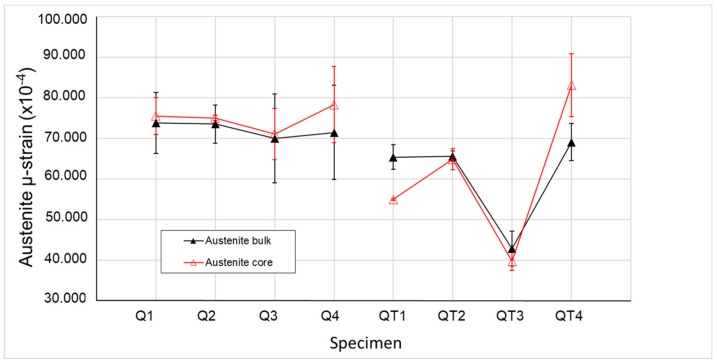
Distribution of µ-strain in FCC.

The evaluation of the reflection profile using an anisotropic strain-broadening model incorporated in the FullProf program (Version 6.30 - Sep2018, The *FullProf* Suite, France) revealed an average and directional µ-strain (combination of strain of type-II and type-III) of the individual phases. Figure 7 and Figure 8 show the values of the µ-strain for all specimens, both for the bulk and the core areas for the ferrite and austenite phase, respectively. A strong directional asymmetry was found for the ferrite phase (µ-strain along 100 is significantly higher than the one along 111, see Figure 7) in comparison with the austenite phase where the asymmetry is minimal (represented by error bars in Figure 8).

Specimens Q1 and Q2, which were quenched under the same conditions until ambient temperature, had comparable values of the ferrite µ-strain. A small decrease of the ferrite µ-strain was observed for Q3 and Q4 specimens, which were quenched to the higher temperature. All correlated tempered specimens QT showed a significant decrease of the µ-strain. An increase for specimen QT4 is due to the lowest tempering temperature 300 °C from the QT specimens. 

The austenite µ-strain is smaller than in the ferrite phase. The values were comparable for all heat treatment samples, except the QT3 specimen where it was significantly smaller. It is in good agreement with the fact that the QT3 specimen had the highest RA content and also the highest hardness (see Table 3) in comparison to other QT specimens. The effect of the tempering temperature on the austenite µ-strain could be described by a comparison of µ-strain values in the core areas: the higher the tempering temperature (460 °C), the lower µ-strain in the core area was observed. Tempering reduced dislocation density and the µ-strain of phases depending on the tempering temperature. The quenched steel has a high dislocation density of about 10^15^ m^-2^, which correlated with the µ-strain values that was found by Harjo et al. [41]. Lower tempering temperature (420 °C) contributed to higher values of µ-strain, i.e., internal stresses due to the dislocations and other intergranular defects, which could be the reason for the lower plasticity of the QT2 specimen in comparison with specimen QT1 (Table 3). An additional reason for the lower plasticity of the QT2 specimen could be tempering embrittlement, which was studied in [20].

Specimen QT3 had the lowest value of austenite µ-strain for both core and rim areas, which could be explained by the combination of initial low µ-strain after interrupted quenching and by following the µ-strain release after sufficient tempering under 460 °C. Sample Specimen QT4, which was similarly quenched but tempered under 300 °C, had higher values of the µ-strain that is linked with the carbon diffusion within the phases. As the carbon activity after heating up to 300 °C was low, the µ-strain value of the sample QT4 could be considered to be very close to the value in the quenched Q4 and in the Q3 specimen, respectively.

### 3.3. Mössbauer Spectroscopy

**Figure 9:**
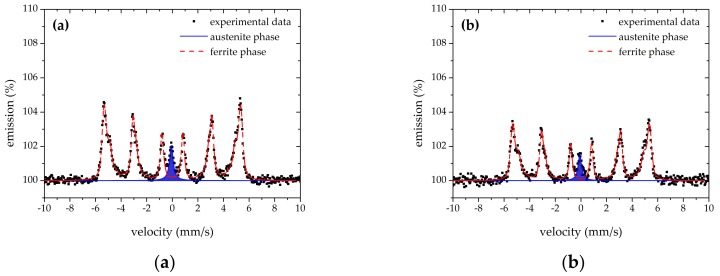

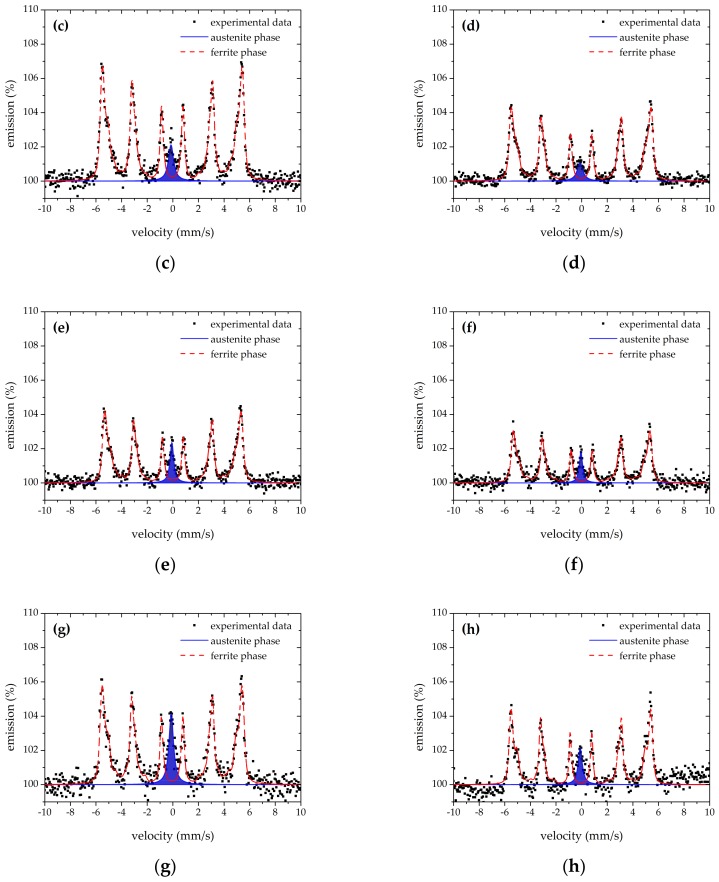
Mössbauer spectra: (**a**) specimen Q1—bulk; (**b**) specimen Q1—rim area; (**c**) specimen QT1—bulk; (**d**) specimen QT1—rim area; (**e**) specimen Q3—bulk; (**f**) specimen Q3—rim area; (**g**) specimen QT3—bulk; (**h**) specimen QT3—rim area.

Backscattering the ^57^Fe Mössbauer spectroscopy results indicates the same shape for all the Mössbauer spectra for the specimens. Figure 9 shows the recorded Mössbauer spectra for specimens Q1 and QT1, as well as Q3 and QT3, (both core and rim) after implemented fitting. The intensity of the austenite singlet lines was decreased for the QT1 specimen in comparison with Q1, which showed the decrease in the amount of RA in both bulk and rim areas.

Selected parameters that resulted from the fitting of the obtained spectra are listed in Table 4. With the amount of RA that is determined by spectral area of the phase in the spectrum (relative to all iron-bearing phases in specimen), the following parameters were obtained and are presented: δ—isomer shift and LW—spectral line width.

Isomer shifts had values of around 0.00 ± 0.07 mm/s. Q samples exhibited the average hyperfine magnetic field B*_hf_ in the interval 31.5 ± 0.5 T, while QT samples exhibited the B*_hf_ in the interval 32.5 ± 0.5 T. No iron carbides (cementite) were distinguished in the spectra, which would exhibit a sextet component with a low *B*_hf_ value of ~20 T.

Only the Q4 bulk Mössbauer measurement exhibited a positive value for the RA isomer shift *δ* = 0.02 mm/s, so the lowest value of the average hyperfine magnetic field B*_hf_ = 31.0 T for the ferrite was calculated. Oppositely, the QT2 rim Mössbauer measurement exhibited the lowest (negative) value of RA isomer shift *δ* = −0.15 mm/s, and the highest value of ferrite average hyperfine magnetic field B*_hf_ = 32.9 T was also calculated. The QT2 rim specimen exhibited the lowest line width value LW = 0.26 mm/s of an RA singlet pattern. Contrarily, the highest LW = 0.55 mm/s of RA singlet was found for the QT1 rim specimen, which could inspire ideas of fitting the RA by a more complex way as a combination of a few singlets and/or doublets [42,43,44,45,46,47]. Hence, the structure of RA in such a case could be discussed. The same recoilless fraction was taking into account for both austenite and ferrite phases [43,45].

### 3.4. Electron Backscatter Diffraction (EBSD) Analysis 

EBSD was the third method applied in this study for the quantitative analysis of the microstructure. The method was based on the collecting of the diffracted patters (the Kikuchi bands) from the focus electron beam in a scanning electron microscope with their following indexing in order to obtain the complex information about microstructure, phase distribution, crystal orientation, and strain within the polycrystalline material.

**Figure 10:**
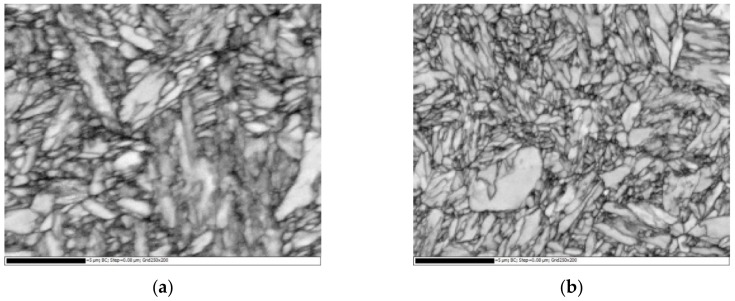

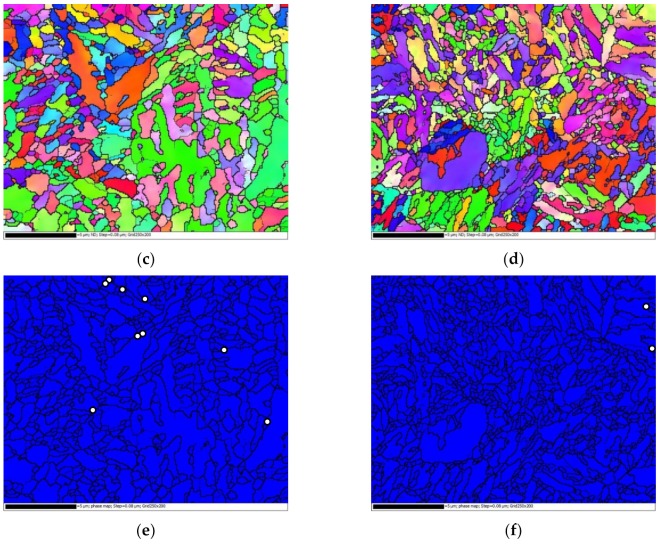
Results of the electron backscatter diffraction for specimen Q1: (**a**) quality image for rim area; (**b**) quality image for core area; (**c**) crystal orientation map for rim area; (**d**) crystal orientation map for core area; (**e**) phase map for rim area (blue—Fe BCC, white dots—Fe FCC); (**f**) phase map for core area (blue—Fe BCC, white dots—Fe FCC).

For all analyzed samples, the fine grain structure was observed. The results of the EBSD analysis for specimen Q1 (both rim and core areas) are presented in Figure 10. The amount of indexed points was on average 70–80% (Figure 10c,d) and the amount of the observed FCC fraction was about 0.01% (Figure 10e,f), which was significantly low in comparison with the results obtained by the means of neutron diffraction and the Mössbauer spectroscopy.

Based on obtained results, it can be suggested that the EBSD technique is limited in the evaluation of the amount of RA. This method cannot be applied in order to evaluate the distribution of RA in high carbon fine grain steels due to the relatively small size of RA films and also due to the high dislocation density in phase boundaries. The same limitations were also observed by Hofer et al. [12] due to their inability to resolve the ultra fine austenite films in the structure.

## 4. Conclusions

In this study, four schemes of the inductive heat treatment were applied for the inductive heat treatment of fine grain vanadium micro-alloyed spring steel in order to obtain the best combination of high strength (above 2100 N/mm^2^) and sufficient ductility. The microstructures were studied by means of three analytical methods, and the main focus was given to the assessment of RA. The analytical techniques of neutron powder diffraction, Mössbauer spectroscopy, and EBSD analysis were applied. and the results can be concluded as following:

- Neutron diffraction provided the results from the bulk of the material. Depending on the quenching and tempering parameters, the measured amount of the RA was 5.8 (2) % for specimens QT1 and QT2, which did not change significantly from the measured Q1 and Q2 specimens, respectively. The amount of RA for specimens QT3 and QT4 was on average 14.0 (2) % because of the interrupted quenching to 180 °C, in comparison with the quenching to 40 °C for specimens QT1 and QT2. Additionally, RA was proven to distribute in the core area of the specimens due to the fast inductive heat treatment, which provides a lower transformation speed in the core area of the specimens in comparison with the surface layer.
- Backscattering the Mössbauer spectroscopy, a commercial newly developed method of RA assessment, provided the results from a polished cross-section surface (depth up to 1–20 µm from the surface) and showed a correlation with the neutron diffraction within the scatter range (see Table 4). Mössbauer spectroscopy also confirmed the excessive amount of RA in the core area of the specimens.
- The EBSD analysis is usually applied in order to describe the phase distribution in the microstructure, and this study deals with the limitations of this method. Although, the average amount of indexed points was 70–80%, the FCC phase was not resolved, which allowed the prediction of the ultra-fine RA films in the microstructure.
- Due to higher content of silicon, RA was stabilized in the microstructure after tempering, which was confirmed by the results of both the neutron diffraction and Mössbauer spectroscopy, and the ultra-fine RA films around the martensitic phase were predicted by the EBSD method.
- Although stabilized RA did not deteriorate the ductility and plasticity of the inductive quenched and tempered specimens QT1 and QT3, a high amount of the soft FCC phase reduced the tensile strength and hardness of the heat-treated steel. Hence, the suggested interrupted quenching for specimen QT3 was found to not be reliable for the production of inductive heat-treated spring steel with advanced properties and a UTS above 2100 N/mm^2^.
- The differences in mechanical properties of the specimens QT after the heat treatment were explained not only based on the amount of RA, but also based on the µ-strain in the BCC and FCC phases measured by neutron diffraction. Specimen QT1 had the best combination of strength and plasticity due to a higher amount of martensite in the structure (after quenching to 40 °C) and sufficient tempering (460 °C), which contributed to the increase of the tensile strength and decrease of the µ-strain, especially in the core area, respectively. Lower tempering temperatures lead to higher µ-strain values and they support the brittle fracture.

## Figures and Tables

**Table 1:** Chemical composition of the steel (in wt.%).

Element	C	Mn	Si	Cr	V	Ni	Mo	Fe
	0.560	0.580	1.400	0.570	0.150	0.024	0.002	balanced

**Table 2:** Parameters of the induction heat treatment.

Specimen	Austenitization Temperature, °C	Temperature after Quenching, °C	Tempering Temperature, °C
Q1	850	40	-
Q2	850	40	-
Q3	850	180	-
Q4	850	180	-
QT1	850	40	460
QT2	850	40	420
QT3	850	180	460
QT4	850	180	300

**Table 3:** Mechanical properties of the specimens.

Specimen	Ultimate Tensile Strength, N/mm^2^	Reduction of Area, %	Average Hardness Value, Method HV1
Q1	-	-	864
Q2	-	-	862
Q3	-	-	780
Q4	-	-	788
QT1	2114	35	635
QT2	2176	19	653
QT3	1815	36	580
QT4	1699	0	692

**Table 4:** Numerical results of the Mössbauer spectroscopy in comparison with neutron diffraction.

Specimen	RA ± 1.00^*^, %	δ ± 0.02, mm/s	LW ± 0.05, mm/s	RA Measured by Neutron Diffraction, %
Q1 bulk	7.10	−0.07	0.35	5.81 ± 0.16
Q1 rim	6.70	−0.09	0.33	
Q2 bulk	6.80	−0.09	0.40	5.48 ± 0.16
Q2 rim	6.20	−0.10	0.37	
Q3 bulk	8.60	−0.07	0.35	7.73 ± 0.16
Q3 rim	8.40	−0.05	0.31	
Q4 bulk	8.60	0.02	0.39	7.22 ± 0.15
Q4 rim	8.40	−0.06	0.33	
QT1 bulk	6.80	−0.14	0.49	5.76 ± 0.17
QT1 rim	6.10	−0.13	0.55	
QT2 bulk	6.80	−0.12	0.41	5.91 ± 0.15
QT2 rim	3.40	−0.15	0.26	
QT3 bulk	12.70	−0.12	0.44	14.13 ± 0.18
QT3 rim	9.40	−0.11	0.39	
QT4 bulk	14.10	−0.12	0.44	13.77 ± 0.19
QT4 rim	12.60	−0.13	0.37	

* The uncertainty of retained austenite measurement is 1%; however, we present the results with two decimal places to highlight differences in measurement and fitting results.
